# Developing cases for an electronic health record simulation and teaching: Team engagement

**DOI:** 10.1111/medu.14981

**Published:** 2022-12-07

**Authors:** Kim Win Pang, Margien Frederique Bakker, Kimberley Joyce Ploegmakers, Stephanie Medlock

## 1 WHAT PROBLEMS WERE ADDRESSED?

Students' first exposure to an electronic health record (EHR) is in our curriculum only at the start of their clerkships. Teaching about retrieving relevant EHR information for clinical reasoning, formulating and documenting a care plan, and communicating this plan to others while simulating a clinical ward was therefore needed. Developing such a simulation and engaging clinicians for teaching was required.

## 2 WHAT WAS TRIED?

We implemented a free and open source EHR in the year 4 curriculum, before medical students enter clerkships.^1^ We instructed internists to write clinical cases with the accordant laboratory, microbiology and imaging results that unfold over time to simulate a medical ward. Cases covered the basics of internal medicine and needed to be aligned with in‐class activities: Analysing information from the EHR, clinical reasoning and hand over skills. A draft version, within 2 months after instruction, was requested, so a medical informatician could transcribe these cases into the EHR. In our program, 24 students start this course with the open source EHR every 3 weeks throughout the year; therefore, a pool of involved internists was formed to teach these classes.

## 3 WHAT LESSONS WERE LEARNED?

A core team of people, including a leading medical educator and an expert with both medical and EHR knowledge, is needed to implement an EHR within 1 year. Parallel to the implementation of the system, the challenge was writing medical cases that complied with the learning goals. A second challenge was ensuring that each case evolved during the simulated admission and that it remained medically consistent. Although we provided enough time for clinical case writing, we found that another 4 months to review the final versions of cases was needed. Providing a template for internists could save time, since all our cases needed reviewing, fine‐tuning and restructuring before transcription into the EHR. Finally, to ensure alignment of the EHR cases with in‐class activities, we strongly recommend regular meetings with the author of each case, led by someone with in‐depth clinical and didactic knowledge to assure this alignment. Considerable time from involved internists is required to teach: they need time to familiarise themselves with the cases, learn the EHR, and establish connection with students. We recommend developing a committed pool of internists who rotate in teaching. Training material for internists can support them during preparation, but they still need the time to review it before class. We provided slides for use during in‐class activities and a short overview of learning goals for each class to minimise preparation time. The immediate feedback from internists during in‐class activities, supplemented with practical tips from the clinic, made students more confident. At the same time, students were motivated and engaged during class since they felt a relationship with the patients from the EHR. To successfully simulate a clinical ward using an EHR for teaching, sufficient time, committed team members and communication between teams is essential. We advocate assigning one leading medical educator to keep the IT team, clinical content development team and educational integrity all aligned. Involvement of a core team of internists for teaching is critical.

## AUTHOR CONTRIBUTIONS

**Kim Win Pang:** Conceptualization, Methodology, Resources, Writing ‐ Original Draft, Project administration. **Margien Frederique Bakker:** Conceptualization, Methodology, Resources, Writing ‐ Review & Editing. **Kimberley Joyce Ploegmakers:** Conceptualization, Methodology, Software, Writing ‐ Review & Editing. **Stephanie Medlock**: Conceptualization, Methodology, Software, Resources, Writing ‐ Review & Editing, Project administration, Funding acquisition.
